# Supplementary material for: Indole produced during dysbiosis mediates host–microorganism chemical communication
Source: eLife. 2023 Nov 21;12:e85362. doi: 10.7554/eLife.85362 (PMC10691800; doi:10.7554/eLife.85362)
Supplement: Supplementary file 1. [file elife-85362-supp1.docx]

**
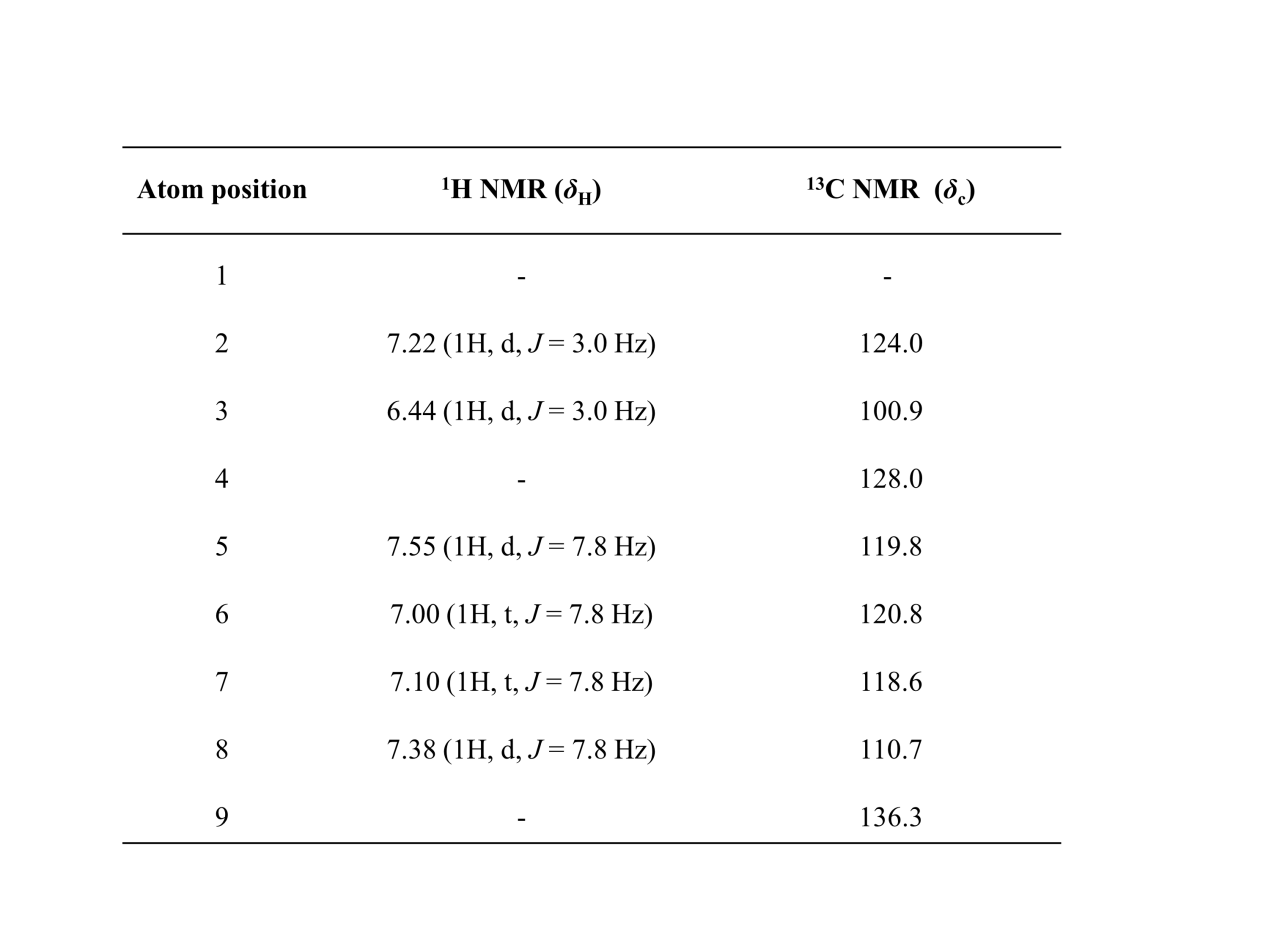
**

**Table S1. The ^1^H and ^13^C NMR spectroscopic data of indole at 600 MHz for ^1^H NMR and 150 MHz for ^13^C NMR with reference to the solvent signals.**

NMR spectra of indole were recorded in CDCl_3_. The corresponding ^1^H and^13^C NMR spectra were depicted in **Figure 2-figure supplement 1**, respectively. *δ*_H_ were recorded at 600 MHz and the measured values of *δ*_H_ were in good agree with published NMR data for indole. *δ*_C_ were recorded at 150 MHz and the values exhibited a good consistency with published data in ppm ([Yagudaev, 1986](#_ENREF_1)).

**References**

Yagudaev, M.R. (1986). Application Of H-1 And C-13 Nmr-Spectroscopy In Structural Investigations Of Indole Vinca Alkaloids. Khim Prirodnyk Soedi, 3-15.
