# Supplementary material for: Indole produced during dysbiosis mediates host–microorganism chemical communication
Source: eLife. 2023 Nov 21;12:e85362. doi: 10.7554/eLife.85362 (PMC10691800; doi:10.7554/eLife.85362)
Supplement: Supplementary file 2. [file elife-85362-supp2.docx]

**Table S2. The data of activity-guided isolation.**

The activity of the candidate compounds isolated from the supernatant of E. coli were measured by the degree of DAF-16 nuclear translocation. These results are means ± SD of three independent experiments. *P*-value < 0.05 was treated as effective. *P*-values were calculated using a Chi-square test.
